# The Poor “Wealth” of Brazilian Football: How Poverty May Shape Skill and Expertise of Players

**DOI:** 10.3389/fspor.2021.635241

**Published:** 2021-03-11

**Authors:** Luiz Uehara, Mark Falcous, Chris Button, Keith Davids, Duarte Araújo, Adelgício Ribeiro de Paula, John Saunders

**Affiliations:** ^1^School of Physical Education, Sport and Exercise Sciences, University of Otago, Dunedin, New Zealand; ^2^Sport and Human Performance Research Group, Sheffield Hallam University, Sheffield, United Kingdom; ^3^Centro Interdisciplinar de Estudo da Performance Humana, Faculdade de Motricidade Humana, Universidade de Lisboa, Lisbon, Portugal; ^4^Secretaria da Educação do Estado de São Paulo, São Paulo, Brazil; ^5^School of Exercise Science, Australian Catholic University, Brisbane, QLD, Australia

**Keywords:** ecological dynamics, skill acquisition, football, poverty, affordances landscapes

## Abstract

Worldwide, 1.3 billion people live in *Poverty*, a socio-economic status that has been identified as a key determinant of a lack of sports participation. Still, numerous athletes around the world have grown up in underprivileged socio-economic conditions. This is the case in Brazil, a country with around 13.5 million impoverished citizens, yet, over decades, many of its best professional footballers have emerged from its favelas. In this article, we explore the role of the socio-cultural-economic constraints in shaping the development of skill and expertise of Brazilian professional football players. The methodological and epistemological assumptions of the “*Contextualized Skill Acquisition Research*” (CSAR) approach are used as an underpinning framework for organizing and analyzing data. Results suggested that, at the exosystemic level of Brazilian society, *Poverty* emerges as an influential constraint that can potentially enrich football development experiences of Brazilian players. *Poverty*, however, is not the *direct* causation of outstanding football skill development. Rather, from the perspective of ecological dynamics, *Poverty* creates specific contexts that can lead to the emergence of physical as well as socio-cultural environment constraints (e.g., *Pelada, Malandragem*) that can shape affordances (opportunities) for skill acquisition. These ideas suggest the need to ensure that environmental constraints can support people to amuse themselves cheaply, gain access to employment opportunities and maintain health and well-being through (unstructured and more structured) sport and physical activities in dense urban environments such as favelas, inner city areas, and banlieues. For this purpose, design of open play areas and even parkour installations can provide affordances landscapes for physical activity and sports participation in urban settings.

## Introduction

Brazil is a country perhaps best known for its Amazon rainforest, Carnival, but in particular, football (see Lever, 1995; Goldblatt, 2006). As Freire (2011) pointed out, Brazilians and football have enjoyed a perfect marriage with successful outcomes such as winning five World Cups and several other triumphs; and for producing many exceptional footballers (see Bellos, 2002; Ankersen, 2013). Unfortunately, Brazil is also known for its challenging social issues such as corruption, inequality, and *Poverty*. Such socio-economic issues have been endemic in Brazil, with data showing that in 1960, the rich, who represented 5% of the population, received 27.7% of national income, rising to 35.8% in 1990. In contrast, the poorest representing 20% of the population received 3.5% of income in 1960, a declining to 2.3% in 1990 (see Eakin, 1998). Today, the number of Brazilians living in extreme poverty is estimated at 13.5 million (IBGE, 2019). Hence, such underprivileged socio-economic conditions (continue to) touch the lives of millions of Brazilian children, including those who later go on to perform at professional level in football.

According to the United Nations (2020a), “Poverty entails more than the lack of income and productive resources to ensure sustainable livelihoods.” Specifically, it is multi-faceted in that “Its manifestations include hunger and malnutrition, limited access to education and other basic services, social discrimination and exclusion, as well as the lack of participation in decision-making.” Thus, *Poverty* is more than solely economic in nature. However, little has been addressed in relation to questions pertinent to the relationship between development of skills and expertise and *Poverty*. This is particularly evident when addressing related questions through the theory of ecological dynamics, which explains the emergence of skills and expertise in sport through the interaction of task, individual, and environmental constraints (Button et al., 2020). At the socio-economic level, *Poverty* may be an influential environmental constraint.

Thus far, broader questions of *Poverty* and sports participation opportunities have largely been approached sociologically, utilizing concepts of social class in Western contexts. This research, whilst understood through multiple theoretical lenses (for a review see Newman and Falcous, 2013), has focussed upon social power relations and inequality. In western nations, relationships have been found between social status and sports participation, identifying higher social class status as offering more opportunities, access and particular attitudes toward physical activity and sport.

In this regard, organized sport is widely understood as a cultural site that is marked by stratified opportunities and widely varying access. The concept of socio-economic status (SES) has privileged understanding of such issues in economic terms. The term “social class,” however, has also been conceptualized in broader terms. For instance, writers have emphasized economic capital (financial assets, wealth) but also identify cultural (qualifications, acquired knowledge, cultural codes, ways of speaking), social (relationships, networks of relationships), and symbolic (honor, status) forms of capital as significant in determining and reproducing social positions and hence in sharpening sports participation. Numerous writers have drawn on the work of French sociologist Bourdieu (1978, 1984) for whom sporting practices are entangled in a continual striving for capital and as sites that reinforce social class boundaries and identities. Thus, he understood sport as a site of “distinction”—with social class significant in shaping opportunities, access, and attitudes toward physical activity and sport. Bourdieu's concept of habitus, which captures those aspects of class-based culture that are anchored in the body or daily practices of individuals, groups, societies, and nations has been influential. More specifically, habitus captures a set of acquired/socially learned habits, bodily skills, styles, sensibilities, dispositions and tastes that can be understood as taken for granted for a specific social class grouping. In Bourdieu's analysis class-based habitus is the result of strivings for class “distinction,” not simply individual preferences. Social class groupings then form themselves by cultivating distinguishing features and signs of “distinction.”

For Bourdieu habitus consists of both the hexis (the tendency to hold and use one's body in a certain way, such as posture and accent) and mindsets, expressed in judgments, appreciation/tastes, and feelings, for example. The concept of bodily hexis may provide a useful conceptual scheme to understand the linkage between the bio-physical body and the socially constituted body. That is, hexis is about more than individual “habits” expressed at the bodily level, instead capturing how the individual body is also collectively and socially shaped. From an ecological dynamics perspective, the concept of hexis can be seen as the symmetric interactive dimension between organismic (body) and environmental (physical and socio-cultural) constraints. In this sense, the sociological notion of hexis can open up questions in skill acquisition terms, especially in relation to linkages between socio-economic-cultural constraints and skill development that may be predisposed by particular social contexts and dispositions. *Poverty* is one such social context.

One reason why the relationship between development of skill and expertise in sport and *Poverty* has not been widely addressed, may be explained in paradigmatic terms. Traditionally, skill acquisition research has often been undertaken from a positivistic, hypothetic-deductive, laboratory-based approach (Uehara et al., 2016). This state of affairs has been called a “significant limitation,” leading to criticisms that skill acquisition theory may be too task-driven, rather than seeking explanations that are based on a broader range of constraints (Newell, 1989). In other words, theoretical understanding needs to be predicated on a wider range of variables related to unique personal constraints of learners interacting with task- and environmental-related factors in the skill acquisition process (e.g., kinematic analysis of hip, knee, and ankle of the kicking leg when chipping a football ball; see Araújo and Davids, 2011; Button et al., 2020 for a review). Such inquiry relies on research tools that are effective when investigating tangible variables that can be measured in a quantitative manner. However, for the study of socio-cultural constraints which are clearly complex, numerous and irreducible, other methods of inquiry are required.

In this sense, Larsen et al. (2013) highlighted the importance of considering the overall environment (i.e., holistic ecological approach) when investigating talent development in sport. They argued that the holistic ecological approach provides methodological tools capable of analyzing not only individual constraints but also environmental constraints such as organizations' settings and strategies.

To shed light on this issue of investigating a broader range of constraints, the Contextualized Skill Acquisition Research (CSAR) framework has been proposed for collecting, analyzing, and discussing data of socio-cultural nature; and in turn for bridging the gap between sociology and sport science (Uehara et al., 2016). Briefly, the CSAR framework is underpinned by the philosophical assumption of the interpretive paradigm, the theoretical principles of Bronfenbrenner's bioecological model of human development, Willis's (2000) ethnographic strategy of inquiry, and the correspondence theory of truth (Dunwoody and College, 2009). It should be noted that, the theory of the ecological dynamics forms the overarching foundation for the implementation of this framework (see Methodology section for further details; see also Uehara et al., 2016 for a review). Ecological dynamics provides movement scientists with a powerful platform to help explain human movement behavior through principles such as self-organization under constraint and perception-action coupling (Button et al., 2020).

Derived from the CSAR, a series of studies has been conducted by Uehara et al. (2018, 2019, 2020) which highlight the relevance of considering complex, interacting socio-cultural constraints upon the formation of football expertise of Brazilian players. Brazilian football is the chosen research vehicle due to its historical tradition of developing high standard football players who seem to emerge from informal, unconventional, and even aversive environmental constraints.

To this end, the present research complements Uehara and colleagues' attempts to answer the following central question: What are the unique socio-cultural environment constraints that influence the development of a distinctive and high caliber of perceptual-motor skills in Brazilian football players? To answer this question, this paper specifically addresses the intersection of skill acquisition in Brazilian football and *Poverty*. In other words, this study aims to investigate the influence of *Poverty* as one of the socio-cultural constraints affecting the development of skill and expertise of Brazilian football players.

## Methodology

This paper builds on a multi-methodological approach underlined by the interpretive paradigm. Through the process proposed by the CSAR framework, Bronfenbrenner's bioecological model was used to organize our data, which were generated via an ethnographic strategy comprising three data collection techniques: contextual analysis, participant observation, and unstructured interviews. Guided by the qualitative analytical steps proposed by Creswell (2009), saturated data were codified and analyzed. The credibility of our findings and analyses have been based on the tenets of the correspondence theory of truth in which the truth is based on a thick description of the variables analyzed through a thorough process of contextualization and reflexivity (Dowling, 2008). Subsequently the discussion and interpretation of the findings are related to key concepts from the ecological dynamics theoretical perspective. Overall, this study was conducted with scientific rigor as well as with interpretive originality supported by the notion of bricolage of the qualitative methods of inquiry. As such, whilst this paper has been structured in the traditional way with abstract, introduction, methods, results and discussion sections, readers are invited to immerse themselves in a dialogical and dialectical process of reading throughout the entire paper so that the key points of articulations that intersect *Poverty* and skill-acquisition can be insightfully interpreted. All procedures were conducted according to the ethical guidelines of the University of Otago Ethics Committee (ref: 10/158) and all participants provided written consent before taking part in the study.

### Contextualized Skill Acquisition Research (CSAR): An Ecological Dynamics Conceptualization

As highlighted previously, the central pillar of the CSAR is underlined by the concepts of the ecological dynamics approach which describes the emergence of expertise in developing athletes as a function of interacting task, individual and environmental constraints (Button et al., 2020). When analyzing intangible variables such as socio-cultural, environmental constraints on sport expertise, Bronfenbrenner's bioecological model of human development (Bronfenbrenner, 2005) offers the scaffolding to help identify and understand emerging data themes. In this sense, the bioecological model is useful in considering human development as a function of the interaction between nature and nurture (Krebs, 2009), that is, between individual and environmental constraints.

Based on a nested scheme, the environmental contexts of the bioecological model are composed of four different, but interconnected, systems including the microsystem, the mesosystem, the exosystem, and the macrosystem (Araújo et al., 2010; Uehara et al., 2016). In relation to each environmental context, only microsystems are physically located (e.g., *Pelada*, i.e., pickup games). The others are “events or forces” that influence the person and the particular microsystem under analysis. The mesosystem encompass other microsystems frequented by the person (e.g., family support, and training system). The exosystem comprises the microsystems that indirectly influence the person and the microsystem under analysis (e.g., a nation economic situation). The macrosystem embraces the overarching patterns of the micro, meso, and exosystems contexts of a given culture (e.g., *Samba, Capoeira, Ginga*, and *Malandragem*). Further than the person and the context, the bioecological model comprises time and process. Process expresses the characteristics of person-context interactions over time. Additionally, person and context change over time (Araújo et al., 2010).

Relevant to our research aim is the exosystem, which refers to one or more settings or contexts that do not involve the developing person as an active participant, but which influence a person's behavior and development. In other words, a child in development is not responsible for the financial situation of his family as neither he/she directly participate in the type of job his/her parents have. However, this financial situation indirectly influences process with the immediate settings for that individual. As an example in football, a lower income family may not be able to provide access to appropriate facilities nor provide adequate shoes for their child. As a consequence, the child has to learn their skill in bare feet in unconventional facilities such as the ones provided in *Pelada* (i.e., pickup games).

However, the bioecological nested system does not operate with clear-cut boundary definitions to classify variables in context (Bronfenbrenner, 2005). It depends on how the context is situated and theorized. In the case of *Poverty*, for example, it could have been classified differently. However, as per this paper, it is categorized under the exosystem context when most likely a family is only poor due to the unequal and corrupted socio-economic system of the country in question, for example, Brazil (see further information in the Results section).

Nonetheless, these interconnected systems inform how the relationship between the person and context are organized under what Bronfenbrenner called “proximal processes,” which change over time (19). The mechanisms underlying the proximal processes “encompass particular forms of interaction between organism [person] and environment…, that operate over time and are posited as the primary mechanism producing human development” (Bronfenbrenner and Morris, 2006, p. 795). However, it is important to emphasize here that, while Bronfenbrenner's bioecological model offers an effective “socio-cultural” framework for the analysis of human development, it does not provide analytical tools required to investigate and interpret processes of skill acquisition (Araújo et al., 2010). For that, related studies rely on the tenets of the ecological dynamics framework to explain socio-cultural constraints on skill acquisition, as explained earlier.

### Ethnographic Strategy of the Inquiry

The ethnographic approach adopted in this study was based on the notion of “*the ethnographic imagination*,” proposed by Willis (2000), who advocated that “… [the] ethnographic imagination is relevant to the production of all kinds of intellectual work. Non-field-based writing and intellectual work [e.g., contextual analysis] can certainly inform the crafts and methods of ethnography” (p. 113). The essence of this inquiry involves practical criticism, rather than mere description; the analysis of lived everyday culture from different sources; and the unique perspective of the researcher.

Three ethnographic methods were employed in this study: contextual analysis (conducted prior to and after fieldwork in Brazil); participant-observation, and unstructured interviews (both during fieldwork in Brazil). These methods are interrelated and complementary in a non-linear, non-sequential research analytical process based on the notion of *reflexivity* described by Dowling (2008).

### Contextual Analysis

The contextual analysis in this paper involves explication of the socio-cultural-economic context in which football in Brazil has been historically constructed (Patton, 2002; Silverman, 2006). In doing so, a number of socio-cultural and political-economic sites of articulation within Brazilian football were elucidated. In particular, these sites of articulation involve the ethos of the white as well as the black and mulatto people in the early years of football in Brazil. Predominantly informed by written texts from sources such as newspapers, articles, books, films, and the internet regarding the history of Brazilian football as well as the broader history of the country, the historical, economic, political, socio-cultural contexts in which acquisition of Brazilian football expertise occurs was significant for this investigation. To facilitate data collection of texts, the first author used a notebook to write notes about the key ideas in the text and the credibility of the data source. His reflections about the document were also recorded.

### Participant Observation

To investigate the topic and generate rich and apt evidence, the first author was prepared to collect data from whatever and whomever provided an opportunity (Patton, 2002; Silverman, 2006), be that from professional or non-professional people related to football, structured or non-structured football settings.

The chosen locations for data collection were based on the parameters of contemporary commentaries regarding the history of Brazilian football, which shows that many successful players emerged from underprivileged suburbs around Brazil (Bellos, 2002). The field research started in Jundiaí—the hometown of the first author—province of São Paulo in two moments in time: first in 2010–2011 and once more in 2017. This location was chosen because of the privileged access that first author had to the place and people from his childhood connections and through his contacts as a former player in this region. Subsequently, four contrasting environments were purposefully identified for the participation and/or observation of football activities. The identified settings were Paulista Football Club, São Paulo Football Club, a football *Pelada* (i.e., pickup game) instigated by a former professional player, and a favela called Vila Ana (see Table 1 for further details).

**Table 1:** Fieldwork settings for the participant-observation method of inquiry.

**Settings**	**Level**	**Numbers of subjects**	**Age range**	**Achievement**
Paulista FC	Professional	40	17–32	Titles: Copa do Brasil, 2005
São Paulo FC	Youth	25	U18	One of the most successful football clubs in Brazil at professional and youth levels. Their youth training center is amongst the top 10 in the world. See https://goo.gl/maps/PJgVDVJQD3qU5fkDA
Pelada	Friends	20	15–55	With few exceptions, the majority of the subjects in this setting were former professional football players
Favela Vila Ana	Children	20	7–15	Children of all levels of skills and gender joined together to play the typical Brazilian football Pelada

Overall, through observation and informal conversations the first author took notes on the behavior and activities of participants. He focused on the experiences and events that happened during the football training and the meaning of the experiences according to the participant's point of view. The first author also participated in the training in all possible ways, for example, setting up the equipment, carrying water for the players, participating as a player if necessary. He recorded descriptive notes in a field log divided into sections such as physical setting, aim of the training, training activities, instructions from the coach, portraits of the participants, reconstruction of dialogues, accounts of particular events, and informal chats. Demographic information about the date, time, and place were also noted.

### Open-Ended Unstructured Interview

Thirteen Brazilian adults with differing football-related backgrounds (i.e., developing players, ex-professional players, coaches, educators, football administrators, and writers) voluntarily participated in this study. Regarding the developing and ex-professional players, whilst not purposefully sampled *per se*, they all grew up under humble economic living conditions. Some were extremely poor. Due to ethical reasons, especially in relation to the principles of protection and confidentiality, the identity of the participants has not been revealed. Participants are referred to by their initials.

In this sense, rather than be limited by interviewing only one specific group (e.g., professional players), many different actor, specifically related to football (e.g., professional and amateur players, coaches, agents, and writers) were interviewed so that the exploration of the topic could be enhanced (see Patton, 2002). According to the literature, a well-performed unstructured open-ended interview enables such an exploration (Denzin and Lincoln, 2005). As such, the first author asked unstructured and open-ended questions, eliciting the views, and opinions of participants. Because of the open-ended nature of this research, the amount of data collection required to make this study coherent was based on the parameters of “*point of saturation*” or the point where new information no longer emerges (Lincoln and Guba, 1985). This is vital because, if the amount of data is insufficient, then important information may be missed, providing an incomplete exploration of the topic. On the other hand, if data were oversaturated, then redundant information would be displayed (Patton, 2002).

### Analytical Procedures

The contextual analysis of this paper aimed to find key points of articulation that link Brazilian football experiences and its socio-economic-cultural formation. Through a point of saturation process (Lincoln and Guba, 1985), it was found that one of the key sites of articulation entangles the ethos of the white as well as the black and mulatto people in the early years of football in Brazil. This broad historical contextual sensitivity was the starting point of the investigation and analyses of other data generated by the two other adopted methods.

In a non-linear fashion, the analysis of the interview and fieldwork methods were guided by the qualitative analytical steps proposed by Creswell (2009). Interviews were transcribed and field notes were typed. Both sources of material were then translated from Portuguese to English. Although the first author was mainly responsible for the translation, a Brazilian academic teacher also helped with the translation. The first author then read all of the transcripts in order to have a general sense of the information and to reflect upon its overall meaning. Next, the process of coding began by organizing the raw material into chunks of text and then separating paragraphs and sentences into categories (e.g., family, training, dance, street-smart, class, economy, “race”) before bringing meaning to the information.

These categories were further explored with additional analysis identifying emergent themes such as *Pelada, Poverty, Ginga*, and *Malandragem*. These themes were then classified according to the nested systems of the bioecological model (noted above), which encompasses four levels: microsystem, mesosystem, exosystem, and macrosystem. In other words, further analysis revealed the emergence of many different interacting constraints (i.e., themes) such as *Pelada* (Uehara et al., 2018) at the micro-level, *Ginga* and *Malandragem* at the macro-level, and *Poverty* at the exosystemic level of the bioecological model. To finalize the analytical process proposed by Creswell (2009), these themes were then described, interpreted, interconnected, and discussed.

It is important to note here that, due to the complexity of each constraint (theme) involved in the analysis, we were only able to primarily analyse *Poverty* in this paper and briefly discussed it in relation to other variables such as *Pelada, Ginga*, and *Malandragem*. There are other interrelated findings at the macro-level, which have been presented in other publications (e.g., see Uehara et al., 2020). As such, we would like to emphasize here that *Poverty* is not the only explanatory factor, but rather one among several, interlinked socio-cultural-economic constraints that influence expertise development in Brazilian footballers (see Uehara et al., 2016, 2018, 2019).

### Evaluation in the Form of the Correspondence Theory of Truth

The credibility of the research study can be enhanced by a thorough contextualization of a phenomenon, in this case expertise and skill in Brazilian football. Subsequently, agreements about how sources of data corresponded to the development of expertise of Brazilian football players were informed by theory and rely on how coherently and consistently we can interpret the findings (see Dunwoody and College, 2009). However, none of the interpretations were assumed to be value-free or un-influenced by the writer and reader's assumptions and background. Moreover, considering the local people's perspective of the phenomenon in question also enhance the credibility (a plural link to reality) of the research. Such a negotiation is what Saukko (2005) calls dialogic validity.

Furthermore, this research has ensured credibility by drawing from the notion of reflexivity. According to Dowling (2008), reflexivity can be described as “…qualitative researchers' engagement of continuous examination and explanation of how they have influenced a research project” (p. 747). With reflexivity in mind, throughout the development of this project we continuously questioned the methodological decisions undertaken so that, if necessary, we could adjust the research focus without detriment to purpose. Under this parameter, the proposed multi-methodological *contextualized skill acquisition research approach* emerged.

### Summary: Researcher Bricolage

Essentially, in order to achieve the aim of this research, the first author acted as a *bricoleur*. In qualitative research terms, a bricoleur draws from multi-disciplinary perspectives, distinct theoretical and philosophical orientations, and various methods of inquiry (e.g., contextual analysis, participant observation, interviews) in order to interpret social phenomenon generated by complex variables, such as those evidenced in socio-cultural studies (Sparkes, 1992).

In effect, this form of analysis requires a multi-qualitative approach that presents suitable methodological and theoretical insights to investigate linkages between socio-cultural environmental forces and cultural and corporeal practices of Brazilian footballers (Uehara et al., 2016). Further, interpretive analyses have to be historically contextualized so that meaningful interpretations of the acquisition of expertise in football in Brazil can be made. To make sense of participants' understanding of how football players in Brazil develop relevant perceptual-motor skills, the first author inductively explored their perceived experiences, views and subsequently attempted to develop a coherent pattern of meanings from their insights.

To summarize, therefore, the multi-methodological approach underlined by the contextualized skill acquisition research framework required a bricolage that intertwined epistemological and methodological concepts from the Bronfenbrenner's bioecological model of human development, ethnography, the correspondence theory of truth (Uehara et al., 2016), and the ecological dynamics perspective.

## Results

### Describing and Contextualizing Poverty as a Socio-Economic Constraint in Brazilian Football

While football has been a symbol of Brazilian success and a source of pride for the people, the same cannot be said about the socio-economic situation of the country, of which a large gap separates the rich from the poor. According to Suneson and Stebbins (2019), Brazil seats in 5th place in a list of the top fifteen countries with the widest gaps between the rich and the poor, and within that, it ranks among the most corrupt countries in the list. One of the major problems caused by such an inegalitarian society is the lack of opportunity for those living in *Poverty*.

Arguably, access to an adequate and effective education system is the best opportunity that a government can provide to its children. In fact, this is one of the UN's 17 sustainable development goals (United Nations, 2020b). However, Brazil's educational system has historically grossly underserved the poor—to the extent that half of the Brazilian population could not read when Brazil hosted the FIFA World Cup in 1950 (the national literacy rate was 44% in 1940, and 49% in 1950; Souza, 1999). Since then, the rate of illiteracy decreased, but today it is still very high with 11.3 million people at the age of fifteen and above who are classed as illiterate (Oglobo, 2019). This shows that the socio-educational system in Brazil has yet to succeed as many children still do not attend school regularly. In fact, education has never been a priority in their lives and this may be explained by McLoyd (1998) who reported that persistent *Poverty* has detrimental effects on socio-emotional functioning, and school achievement.

Public schools in Brazil struggle with the lack of government support and hence poorly qualified teachers, and substandard facilities, equipment, and security. They are only slightly better because of the voluntary help of community and non-governmental organizations. In contrast, the quality of private schools is far superior. However, with a cost that is almost the monthly salary of a working class person, attendance in private schools is only accessible for the middle and upper classes (Redação, 2020). Consequently, these upper-class students are the ones who tend to go to the best universities in the country and subsequently get the best jobs. And so, the *Poverty* cycle continues with the working poor struggling throughout their entire lives without realistic opportunities for improvement.

With this in mind, football in Brazil emerged out of irreconcilable differences between the rich and the poor. The early clubs were founded within the elite social groups of Rio de Janeiro and São Paulo and played under the English imported ethos of “amateur spirit” where values of chivalry and fair play were paramount to their existence (Guterman, 2009). Hence, football games were almost outdoor parties played for the pleasure of camaraderie, a spectacle of colonial class, status, and racial whiteness. As such, football participation was restricted to people of a similar social and racial background (see Franco, 2007; Priore and Melo, 2009). In this sense, the elite were more than just proclaiming moral values as if it was part of their status, but they could also distinguish themselves from what they saw as the customs of the uneducated immigrants and former slaves (Guterman, 2009).

Despite the initial resistance of the elite strata of society in Brazil, football was soon diffused amongst the masses (Guterman, 2009). However, while the higher social class players had financial power to play under the best facilities such as on grass fields and with specialized coaching, the lower socio-economic classes had to play with bare feet on streets full of stones and mud, and were forced to make their own football materials like goal posts made of bamboo sticks, balls made of socks, and their own rules (Filho, 2003). More than 100 years later, many Brazilian children are still playing under similar penury conditions.

In the fieldwork at favela Vila Ana^1^, the first author had the opportunity to observe and play football/futsal with local youth and teenager players. The venue was a deteriorated futsal court partially built up with money from the “lords” (a term used by the children to refer to the drug dealers). The indigent characteristics described above were present at all levels, including for instance, playing with bare feet, mixed age and gender, using an old, tattered ball, and players self-organizing into teams. However, a characteristic enthusiasm to play, the happiness and celebration of scoring goals, the determination to win, the teasing, and the arguments between players were also evident. In essence, they were playing under the typical spirit of *Pelada* (i.e., pickup games; see Uehara et al., 2018) or in the word of Freire (2011), street football, as further delineated below.

There, the first author informally asked some of the children about their professional football aspirations and frequently their answer was about playing football for the love of the game, but also to improve their socio-economic status. Understandably, by seeing those Brazilian football superstars who made it to the top these children want similar lives too. As two children explained:

Child A: I would like to be a professional football player to make enough money so I don't need to get involved in this kind of life style of using or selling drugs. My whole family played football, including my father. I love it.

Child B: I love football. I would like to be like Ronaldinho. He is my hero!

Under this context, it could therefore be argued that football in Brazil offers opportunities that underprivileged children do not usually have through other means. It is an opportunity for economic independence and social recognition as exemplified by many of the Brazilian football icons such as Pelé, amongst others. They become the heroes of a nation “who represent the triumph of men from a poor background over the wealthy and powerful” (Miller and Crolley, 2007, p. 20). They are the heroes who represent nationally and internationally the history, the values, and the identity of Brazil.

On a parallel but relevant note, the opportunity for economic independence for underprivileged children through football is not only the privilege of Brazilians. Numerous football heroes from other nations have also emerged from *Poverty*, such as the case of Diego Maradona from Argentina. Without getting into the traps of futile comparisons between Maradona and Pelé, *Poverty* was a significant part of their childhoods. Certainly, they both played a lot of street football, and they both achieved incredibly high standards of perceptual-motor skills (see Nascimento, 2006; Maradona, 2007). However, whilst Pelé managed his career off-field as good as on-field, the same cannot be said about Maradona. According to Enkvist (2010), the key point of difference in this respect is, arguably, related to values learned through education. In one hand, there is Pelé, an individual who completed a tertiary degree in physical education and surrounded himself with responsible people. On the other hand, there is Maradona, a person who barely finished the first year of secondary school and many of his support clan were not necessarily people with the best interests (Enkvist, 2010). For instance, when Maradona was transferred to Barcelona in 1982, he brought with him from Argentina a whole group of people, made up of family and friends, who lived with him. They were his personal assistants. Commentators alike use words like “parasites” or “pirates” to qualify them. In order to continue living off the footballer, they flattered him. So much so that Maradona lived immersed in what has come to be called “*sidieguismo*,” meaning: “Yes, Diego” (Enkvist, 2010).

From a skill acquisition point of view, it can be said that Maradona, Pelé, and of course, many other former and current players, were highly influenced by what Freire (2011) refers to as the pedagogy of “street football.” According to Freire (2011), street football offers opportunities to learn and enhance skills in an informal and natural way, emphasizing many important pedagogical principles (e.g., co-teaching, collaborative-learning, modeling, fun and enjoyment, freedom, creativity, improvisation, skill adaptation, challenges; for an overview see Renshaw et al., 2019) that positively shape their experiences. In a similar line of focus, Machado et al. (2019) also highlighted the importance of street football to players' skills development. However, from a socio-educational point of view, as explained by Freire (2011), street football can also be cruel and susceptible to undesirable and detrimental experience and influences, such as lack of inclusiveness, empathy and compassion (e.g., the best players are selected first and the less or non-skillful ones are only chosen to complete the teams). Very often, these non-skillful players are stigmatized and humiliated, and a popular name for them in Brazil is *Perna de Pau* (i.e., wooden leg). In addition, without formal rules or officials to enforce them, street football does not necessarily encompass principles of moral and educational values (Freire, 2011).

To this end, these accounts lead us back to the notion that *Poverty* can *directly* affect in a negative way other socio-cultural constraints such as education. Yet, *indirectly*, it potentially positively influences individual constraints at perceptual-motor skills and expertise levels.

### Poverty as an Exosystem That Can Enrich Football Expertise

An exosystem is an environmental influence which affects a developing person but they are not directly responsible for it. A typical example of an exosystem is the family economic situation in which a child relies on the parents for their upbringing. The economic status of a family may impact the child in either negative or positive ways (see Bronfenbrenner, 1979; Krebs, 2009).

In the case of Brazil, *Poverty* has negatively impacted developing children in a multitude of ways. Besides the schooling issues, as explained above, many children have to get into the informal and very often underpaid working force at an early age to help with the household expenses. Unfortunately, many of these children are easily seduced by the life of crime and illegal drugs. An example is RD an interviewee who explained how he went through this pathway:

Yes, I was poor living in a shanty town and drugs were ‘in my face’ all the time. I tried to avoid it but due to the frustration of living in such conditions plus the pressure from peers, illegal drugs such as crack became part of my life. But there is always a way to overcome it and move towards a healthy lifestyle regardless of socio-economic status. I found my way. I got a degree in physical education and now I coach underprivileged kids from shanty towns in an attempt to guide them for better choices in life. As a physical educator, I am amazed by the level of skills of some of these kids. Everything seems to be natural for them. They never had a formal type of coaching but when they play football, their talent flourishes in the field (Interview, January 13, 2011).

However, not many citizens possess the will power demonstrated by RD to overcome such aversive living conditions. Now he serves as a role model in his community for those underprivileged children to follow suit and at the same time, he can identify and support those with the potential to pursue a football career. As RD pointed out, football skills seem to be common among many of the children from his shantytown. In this regards, JPM—a former Brazilian football national team fitness coach who also dedicates part of his time to voluntarily work with underprivileged children—highlighted this issue as follows:

I think the poorer the child the richer he/she will be in terms of body coordination movement. I don't want to close this information or generalise it, but from my experience as a physical educator and as a coach, I have observed it. In contrast, children from families with financial stability tend to be less physically coordinated, especially in the last fifteen or so years due to the advance of technology, computers, television, and electronic games. They play fewer of those kinds of games the poor children play in a natural learning environment. They tend to spend more time at home. In contrast, children of lower socio-economic status tend to be less educated compared to the middle/rich class children. They tend to focus less on education and like being outside playing. For that reason they are better physically coordinated children. As such, for those poor children who play football they tend to be more skillful players too, comparatively speaking (Interview, February 10, 2011).

As this quotation suggests poor children may be more skillful because they focus more on playing football compared with rich children who tend to have other duties and hobbies. However, other influences have to be taken into consideration when investigating Brazilian football skills as a product of *Poverty*. For instance, it is often the case that children from *Poverty* stricken favelas do not have enough food on a daily basis. Many have parents who are unemployed and possibly are themselves drug users. Thus, inspired by their football heroes, as explained above, many of these children are motivated and determined to get out of these miserable conditions through football.

These ambitions are not new. Since football turned professional in the 1930s and became a national sport, these lower social economic status players have seen football as an achievable way of escaping *Poverty*. As Didi, a Brazilian football superstar in the 50s, argued, “the boy who has an easy life doesn't have a chance in football because he doesn't know the value of a plate of food” (Pelé, 2008, p. 47). This assertion is reinforced in the words of VL:

“When I went to SPFC at the age of fifteen I was feeling like I was walking on a cloud. However, I must say that it was difficult to be on my own. I missed my family and friends a lot. On the other hand, I knew that it was the opportunity of my life. My mom was deeply sad when I left but I tried to cheer her up by promising this: “Soon I will be able to buy you a house”. Years later when I got the money from my first contract, the first thing I did was to keep my promise to her. Subsequently, along the years I bought a house for each of my brothers and sisters. But you see, I had determination and motivation to overcome any obstacles because I knew how hard the dark side of life is when you don't have enough food on your plate. It is quite rare to see middle class players achieving what I have achieved in football. For instance, my son was quite a good footballer, so he was accepted to be part of the SPFC youth academy. Like in my youth days, he had to live in the dormitory of the club which wasn't as near as flash as it is now. Today the training centre in Cotia where the youth players stay is a world class place. But do you think he managed to stay there? No, he couldn't stay there for more than two months. He had to come back to the comfort of his home, even though he knew that by doing so the dream of following the footsteps of his dad was over” (Interview, February 16, 2011).

This quote helps to explain why many football players in Brazil come from lower socio-economic status (see Dana, 2013). Players like VL's son have more options to successfully do well in life than merely by the means of football. As a result, they do not have the motivation and determination for what it takes to become football professionals. In this sense, this quote highlights how resilience is a key psychological virtue, which may be promoted through under-privileged living conditions.

In further discussing this issue of *Poverty* with OA, he offered a controversial point of view that is worth highlighting. In his view:

Now, the better players are those from financially poor families. They are much more skillful and bold too in football. But I ask myself why? This is because they don't have rules at home. They go to other people's house and don't have manners. They act as they were at their own houses. They are not educated to be politically correct. So, as football players, when they go to play away, they do the same, that is, they play as they are playing at home. In my view, they are much more mentally stronger. The thing is, the poor children have so many other difficulties in life that when they play football they don't choke, they play like they are playing football for fun, regardless of the pressure. I say this based on my experience that I have acquired along my career as a player as well as a coach (Interview, February 9, 2011).

As it can be seen, OA was quite radical in his thoughts about the reasons for poor children being resilient. He associated the idea that *Poverty* is synonymous with bad manners and, in turn, with being mentally stronger. Regarding the former (i.e., the relationship between idiosyncratic mannerism and poverty), it is beyond the scope of this article to elaborate further. However, regarding the relationship between *Poverty* and mental strength, further discussion is warranted as it may add important value.

For instance, Emerson, also popularly known in Brazil by the nickname Sheik, was born and raised in a slum in Rio de Janeiro. As a (former) professional footballer, he reached his “glory” by winning, as a decisive player, three consecutive Brazilian Championship titles followed by three different teams: Flamengo, Fluminense, and Corinthians, respectively. For the latter, at a press conference after the final match of Libertadores Championship 2012 against Boca Juniors FC, he explained how life in the favela helped him not to feel the pressure of decisive games. He said:

“I was born and raised in a very simple place, and I saw things that maybe many of you [journalists] will never see. Previously, I was asked if there was pressure to play at the Bombonera stadium in Argentina [first leg game]. Dude, pressure is lying in bed being afraid that stray bullets may hit your face, your chest, yes this is pressure. Playing in a packed stadium with new balls, perfect grass, etc, there is no room to feel pressure, it is all about enjoyment” (Laurentiis, 2012).

Here Emerson explains the relativity of pressure on a football field compared to the violent environments he had faced. Indeed, Emerson did not choke in any of these decisive games. At the Libertadores Championship, he was one of the key players in the first leg game played in Argentina, including setting up the goal Corinthians scored to secure a 1–1 draw. Further, in the second leg game in Brazil, he scored the two goals that made Corinthians the champions of South America. Later in the same year (2012), Corinthians defeated Chelsea 1–0 to win their second FIFA Club World Cup.

The first author's fieldwork at favela Vila Ana can also provide relevant insights on this issue. For instance, he noticed a much younger and smaller boy facing up to the bigger one in an argument. This shows that children learn quickly to stand up for themselves to be able to survive in this kind of environment. From a sociological point of view, this issue may be explained by the notion that *Poverty* intersects with gender, and gender with sports. In this sense, masculinity equates with being strong and fearless which seemingly gives it greater credence and which in turn elevates masculinity to a hierarchical status in football (see MacLean, 1999). From a psychological point of view, it can be argued that being fearless is about developing resilience and a certain mental toughness (see Rachman, 1984).

### Poverty and Unconventional Football Practice Environment: Multi-Interactive Constraints

As highlighted so far, many Brazilian children live in *Poverty* and/or in rural areas and therefore have to draw upon whatever possible physical means to be able to play. On this note, successful Brazilian football players are often associated with the notion of developing their skills in natural learning environments under multiple tasks and environmental conditions (Araújo et al., 2010; Uehara et al., 2018, 2019). Not all Brazilian children who have learned football in an informal natural learning environment were poor. However, the children from poorer families tend to be more exposed as they often live in underdeveloped areas such as favelas or in rural areas that lack structure, infrastructure and adequate educational system. By being exposed to informal learning conditions, many children in Brazil tend to explore more than football itself with other activities such as climbing trees, swimming in lakes, and other physical activities that tend to be challenging yet fun (see Table 2), although with certain restrictions today due to increasing urbanization. Such activities encourage creativity, improvisation, adaptive skills, and ultimately the overall development of perceptual-cognitive-motor skills (see Louv, 2005). Exposure to a range of outdoor environments and opportunities to adapt to dynamic constraints has been recently recognized within the ecological dynamics framework as an important means to promote lifespan skill development (e.g., Rudd et al., 2020).

**Table 2:** Quotes from the interviewees^*^ highlighting their experience on playing in a natural learning environment.

OA	I lived in a small town so we had a lot of space to play and at that time it was safe to play around my neighborhood. We swam in the rivers, climbed trees to get fruit, played hide and seek, etc. Football of-course was my favorite. It was normal for us to play football bare foot with homemade balls.
CL	In think we Brazilians learnt skills in a natural way or at least used to. This helps in the acquisition of skills rather than in just learning tactical movements. Therefore, the fact that I played a lot in a natural environment under all sorts of fun tasks, all of that have positively affected my motor-perceptual skills.
VL	I played football everyday on the street, but I also did what other kids in my time used to do. We trespassed into some farms to get fruit from trees, such as avocado and orange. We learned how to swim in the lakes around. We had to be smart to not come home with wet pants, as if so our mums would smack our bums.
DB	Given that I lived in Rio de Janeiro which is surrounded by hills and mountains, we had a lot of natural environment to play all kind of games, but ultimately we all ended up playing football more than anything else every day on the street.
MS	No doubt that I played more football than anything else. It was and still is my passion. But as a kid I lived in a suburb surrounded by nature and there we were able to play all kind of other games too.
JS	Swimming in the rivers, stealing fruit, running here and there, running after balloons, my childhood was like that. Today that does not exist anymore, well at least in São Paulo city where urbanization has dramatically increased.

* *These interviewees have been directly involved with professional football*.

Underpinning these quotes in Table 2, our interviewees commonly reported that they used to explore different forms of physical activities for fun and enjoyment, which in effect, resemble the practice of *parkour* in a sense of activating all sorts of perceptual-motor and cognitive skills at gross and fine neuro-muscular levels. Briefly, parkour is a sport where practitioners (i.e., traceurs) transverse man-made or natural obstacles with the use of simple and complex actions such as running, climbing, vaulting, jumping, landing, rolling and other movements in order to achieve a talk goal of traveling from one point to another in an innovative and efficient manner (Aggerholm and Højbjerre, 2017).

Still, underpinning the quotes in Table 2, in spite of the other physical engagements, football seems to be the preferable activity for the interviewees. On this note, it has been reported that more than the 11-a-side regulation form of the sport, various other configurations of the game such as *Bobinho* (i.e., rondo), *Rebote* (i.e., rebound), and *Artilheiro* (i.e., striker, top scorer) have composed the traditional culture of playing ball games with the feet (Scaglia et al., 2021). In addition, street football, beach soccer, and futsal have been traditionally played within the Brazilian society (Uehara et al., 2019). In effect, playing under such informal conditions can be linked to other socio-cultural constraints at the micro and macro levels of the Brazilian football such as *Pelada* (i.e., pickup games) and *Malandragem* (i.e., street smart, cunning, trickery, creativity), respectively (Uehara et al., 2018, 2020).

According to Uehara et al. (2018), *Pelada* is a type of spontaneous and unsupervised “pick-up” football that can be played in different physical environment constraints such as the waste grounds and landscapes, streets, schools, beaches, and backyards. Importantly, *Pelada* can be played with very few resources or supervision (i.e., referees, coaches) which removes barriers that other forms of football may present to those living in *Poverty*. Playing in such informal contexts provides the opportunity for the development of high caliber of perceptual-motor skills, including the *Malandragem* skills for deceptive and creative actions so that the game can flow with a *Joga Bonito* (play beautiful) style. That is, the Brazilian *Ginga* (i.e., body sway) style (see Uehara et al., 2020).

In this regard, Mr. VL provides an enlightening comment in which he intersects key points of articulation at different systemic levels such as *Pelada* for microsystem, *Poverty* for exosystem, and *Malandragem* for macrosystem. He says:

I lived in a very rough neighbourhood full of crime. We played *Pelada* every day on the streets. We had players at various levels of skills and age. I was about 6 years old. So everybody knows that football has 17 rules, but in our street there is only one rule: if no blood no foul. Under this context you create certain *Malandragem* [trickery] for the rest of your life. For example, I knew that if I bumped into a 15 year old boy I would break myself up, so I had to look over my shoulders all the time and anticipate the moves to avoid physical contact. In doing so, you develop quick thinking and the notion of searching for space and time to play (Interview, February 16, 2011).

In this sense, Mr. VL shows how the task of playing *Pelada* in an aversive environment constraint shaped his *Malandragem* skills for the purpose of self-preservation. In other words, VL connects the notion of *Malandragem* with key elements of skill acquisition such as anticipation, rapid thinking, perceptual information, decision-making, problem solving, and exploration of space and time.

Furthermore, under the scope of *Malandragem* skills the text below provides another point of articulation in which the mischief/trickery of others creates an opportunity for VL to develop perception and attentiveness skills in relation to other parameters in the playing field:

I was the youngest, my father was killed when I was a baby and my mother was a cook at the neighbourhood school. She worked 14-16 hours a day. So we were very poor. My mom used to make our shorts out of those big cloth bags of sugar. It was the biggest reason for mockery. Sometimes when we were playing football on the street my mates tried to lower my shorts down because I was not wearing underwear. So I had to stay alert all the time looking around (Interview, February 16, 2011).

Here it is interesting to note that *Poverty* and *Malandragem* represented by the notion of perceptual skills are all entangled in a “non-linear fashion.” That is, whilst VL's friends were *Malandro* (i.e., streetwise person, naughty) by being cheeky in trying to lower his unconventional home-made shorts and make fun of him, VL used his *Malandragem* skills to constantly scan his environment and thereby improving his perceptual awareness.

An additional point of articulation provided by VL during the interview is worth highlighting. For him, *Malandragem* is about using *Ginga* (i.e., body sway) to deceive the opposition with body movement that sways from one side to the other. Hence, VL articulates *Ginga* and *Malandragem* all together under the notion of body movement associated with perception, decision-making and ultimately improvisation:

In my view, *Ginga* is synonymous with improvisation. I don't know if you think like me, but when you see someone playing we can say he plays with *Ginga* or not. But *Ginga* is not only about the way that one executes movement, it is also about astutely perceiving what is going on around. It is about being smart and cunning enough to anticipate what is going to happen and make decisions accordingly [Malandragem]. Therefore, based on these parameters I can say that *Ginga* is synonymous with improvisation (Interview, February 16, 2011).

From a socio-cultural point of view, it can be argued that VL's statement represents the typical “malandro” who is “smart and cunning enough” to find rapid solutions in different game situations. Essentially, for the Brazilian, *Ginga-Malandragem* is the utmost skill of perceiving, acting, creating and improvising in sport (for further clarification on this issue, see Uehara et al., 2020; and/or the movie entitled “Pelé: Birth of a Legend” directed and written by Zimbalist and Zimbalist, 2016). Arguably, the iconic Pelé best represents and endorses the Brazilian football *Ginga* style.

To this end, from a skill acquisition point of view, whereas some might have previously perceived the blend of constraints identified in this article as negative or aversive to overall learning and development, in fact much of the data have suggested otherwise. However, it is noteworthy that the pathways to reach success is extremely competitive and many children are left behind. Hence, from a sociological perspective, we can never ignore the fact that *Poverty* is one of the major constraints that negatively affect the development of many children in Brazil and around the world due to the lack of opportunity and economic means to participate in sport (see Newman and Falcous, 2013). In fact, it is common to hear stories in Brazil of talented young players who gave up pursuing a football career due to financial difficulties in their family—e.g., to pay bus tickets to attend training sessions; not having enough food to eat or because of an injury which demands surgery but there are no adequate resources for it (Simas, 2004). These effects of *Poverty* are still around in this millennium and not only in Brazil. Manchester United striker Marcus Rashford reported the same problems in having the money for bus rides and food to eat. Luckily, a coach used to pick him up and drop him home because he was such a talented child (BBC, 2020). However, not all talented children have the same fate.

Therefore, be it for health and/or for performance purposes, it is essential that Government and institutions alike, at all levels, provide means for *all* to participate in sport and physical activities, as further discussed below.

## Discussion

Traditional sociology of sport, whilst not addressing the development of expertise or skill, is of contextual relevance in capturing the constraints associated with social class status. This sizeable body of research literature largely approaches the key environmental influence of low social class status (as an indicator of [relative] *Poverty*) as a *constraint* on sports' participation and access, and hence opportunities for skill acquisition/expertise development. From an ecological dynamics perspective, through the multiple lenses of the CSAR approach, in this article we have examined *Poverty* as one of the socio-economic constraints that *indirectly* influence the development of perceptual-motor skills of Brazilian football players. More specifically, we have provided interpretative analyses of the contextualized sites of articulation to explain the points in which experience of *Poverty* intersects with processes of skill acquisition.

Many children in Brazil live in rural areas and/or in *Poverty*, and therefore, have to draw upon whatever physical environment resources they have to play. However, from a skill acquisition point of view this is not necessarily a degrading constraint as these conditions actually favor the development of their perceptual-motor skills in different sports, like football. In other words, lack of resources can make you resourceful as an individual to explore what, how, when, and the locale for opportunities to play. This is because children' fledgling skills may be shaped in a positive way when playing freely under different environment and task constraints (e.g., climbing trees, swimming in the lakes, and playing hide in seek in the bushes; Uehara et al., 2018). Having had fewer opportunities for education and coaching and more emphasis on playing *Pelada*, these children need to be skillful and creative to succeed in football. As Uehara et al. (2018) pointed out, this informal way of playing football in Brazil often self-organizes on irregular surfaces, played with bare feet, and on small and deteriorating playing spaces and surfaces. However, such aversive task and environmental constraints can actually be beneficial for exploration, discovery and effective learning, and in turn for the development of a high level of perceptual-motor skills (Uehara et al., 2018). Such practice conditions are also known to promote degeneracy in skilled athletes which force them to adapt and recruit multiple movement patterns to satisfy the same task goal (see Seifert et al., 2013). This general argument is also in line with the notion that talented athletes' need challenges to overcome in order to develop characteristics to reach and stay at the top of their sport (Collins and MacNamara, 2012; Collins et al., 2016).

Further enhancement of Brazilian players' perceptual-motor skills can be influenced by other related socio-cultural constraints encountered in the Brazilian society such as *Malandragem* and *Ginga* (Uehara et al., 2020). Ultimately, the entanglement of physical, as well as socio-cultural, constraints such as *Pelada, Poverty, Ginga*, and *Malandragem* serves as a framework that supports the development of skills that go beyond motor and perceptual attributes. In other words, shaped by different environment and task constraints, Brazilian footballers, arguably, develop self-regulatory skills such as emotional control, skill adaptation, resilience and mental toughness to play well anywhere and under any circumstances (Araújo et al., 2010). In addition, the underprivileged living conditions may develop psychological fortitude and motivation to improve their socio-economic status. However, we acknowledge that this aversive constraint shall not be considered the only contributing factor toward talent development because a blend of aversive and coercive constraints is necessary to develop world-class players (see Collins and MacNamara, 2012). In Brazil, for instance, talented players are usually selected to join a federated club by the age of 14 years old, and once there—subject to the level of the club—they receive the professional support and structure to fine-tune their development. Without the input of trainers, specialist coaches, nutritionists, and doctors at some point it is unlikely they would make it to the very top (see Bettega, 2019; Thiengo, 2019).

Using the CSAR rationale, it is important to re-iterate here, therefore, that football expertise development is not directly caused by *Poverty*. Rather, *Poverty* produces specific contexts that, in turn, generate physical as well as socio-cultural environment constraints (e.g., *Pelada, Malandragem*) that can sculpt affordances (opportunities or invitations) for skill acquisition. In this regard, the perspective of ecological dynamics creates a powerful theoretical lens through which one can interpret these empirical observations. Somewhat counterintuitively, the lack of resources that may be denied for children living in poverty (e.g., ball, clothing, pitch, coaching, etc.) may help promote a resourcefulness in their character to overcome this inequity in status. Many of the interviewees we spoke to, confirmed their belief that opportunities to play informally in outdoor settings had been a significant contributing factor in their skill development. As evidenced by a vast number of empirical research studies, the development of expertise in sport, including football, emerges from the interaction of key constraints (Button et al., 2020). In the case of Brazilian football, these constraints can be exemplified as the task (e.g., *Pelada*), organismic (e.g., football players), and environment (e.g., socio-cultural ones such as *Ginga-Malandragem*) constraints (Uehara et al., 2018, 2019, 2020).

### Limitations and Research Recommendations

It is worth noting that not all Brazilian players play with *Ginga*, neither all come from socio-economically underprivileged backgrounds. Kaká for instance, a former football player who won the Fifa player of the year in 2007, comes from a middle-class family and, arguably, his style was closer to the pragmatic European football style rather than Brazilian. On the other hand, Sócrates, a former Brazilian national team player, epitomized the *Ginga* style, yet, he also had a middle-class background. An interviewee (DB) who also grew up in a middle social class family can further add light to this issue. He said:

Growing up in Rio de Janeiro, I used to play with my mates of the same social class, but the boys from the favelas used to come down from the hills and play with us. There was a visible difference in their skills compared to ours. They were much, very much more skillful than us. They had what we say is the essence of Brazilian football and played with trickery, flamboyance and style. Everything that I learnt in terms of football was not from my mates but from those boys some of whom didn't even have enough food on a daily basis. But they were good at football (Interview, December 12, 2010).

Here, DB reveals the difference in skills between poor and rich children and how the former influenced his football skills development. Under this context, a plausible reason for rich Brazilian children developing their *Ginga* style can be explained by what is known in motor learning literature as observation and transfer skills. This is because *Ginga* football style has been so deep rooted as a popular culture that regardless of socio-economic status, children learn from each other by playing with each other, by observing each other carrying therefore the legacy of *Ginga* across generations. However, to further elucidate and contemporize this issue, future research may consider investigating Brazilian football players who were not poor in the first instance. It is possible that future Brazilian footballers will come from a broader spectrum of backgrounds that they have in the past due to the widening gap between the rich and poor.

We acknowledge that funding resources were limited and as a result, fieldwork data were mainly collected in the region of São Paulo city where the first author grew up playing football. However, as per the contextual analysis, the development of Brazilian football players is not limited to one region, but extends to Brazil as a whole. In addition, we are mindful that the skills and expertise of Brazilian footballers are not solely due to an informal play (e.g., *Pelada*), neither are they just a function of aversive environmental constraint such as *Poverty*. Supportive and coercive environmental constraints, such as the ones encountered in family settings and federated clubs play an important role on the development of skill and expertise of Brazilian footballers. Thus, this issue also warrants future research (see Salmela and Moraes, 2003; Bettega, 2019; Thiengo, 2019).

### Implications and Policy Recommendations

In highlighting the issue of *Poverty*, the point of interest is not about being poor to be able to play with *Ginga*. Regardless of social class, the focus should be on the development of a national football training programme with methodology that preserves the essence of Brazilian football style and at the same time makes it better by adding educational values to it. This issue has been addressed by our interviewee JPM:

The number of soccer schools in São Paulo has increased dramatically in the last 20 years. This is due to urbanisation, which is taking away children's natural space for playing. As such, business minded-like people saw the opportunity to open soccer schools so children can continue to play football. However, the problem is twofold: the first is that not all children can afford to pay soccer school fees; the second is that the majority of these soccer schools are not methodologically prepared to coach children, and thus rather than developing them these soccer schools are in fact inhibiting children's football development skills (Interview, February 10, 2011).

In a similar line of focus, ACS said “these emergent soccer schools are inhibitors rather promoters of skill development. That is, they do not have the right methodology to train our children to become good footballers in the future” (Interview, February 8, 2011).

Indeed, this is an important argument to be considered as, arguably, it is notable that there are fewer current exceptional Brazilian football players than there were in previous national teams. Just as an example, in 2002, the last time Brazil won the World Cup, the squad was composed of players such as Ronaldo, Rivaldo, Ronaldinho, and Kaká (all FIFA World Player of the Year award winners), as well as other talented football players such as Cafu and Roberto Carlos (CBF, 2020). Further, most recently the *Seleção* (i.e., the Brazilian national team) performed below the huge expectations of the fans in the 2014 World Cup in Brazil, and 2018 in Russia. In previewing this issue, Rivelino raised concerns about the future of *Seleção* as he claimed that the problem was due to the lack of practicing street-football (i.e., *Pelada*; BBC, 2006).

Despite of all that, paradoxically, Brazil continues being one of the biggest exporters of professional football players worldwide (GloboEsporte, 2020). However, this does not suffice for a country with a strong historical tradition in football. Therefore, to effectively address this issue, it is crucial that Brazilian football organizations understand the importance of the socio-cultural contexts in which football has successfully evolved throughout the history of Brazil. In doing so, the problem of urbanization that has occupied the free spaces for *Pelada*, for example, can be overcome by setting up training centers with qualified coaches that understand the effect of physical as well as socio-cultural constraints influencing the development of perceptual-motor skills of Brazilian players. As JPM pointed out, the key for the success of Brazilian football in the future is to bring back the essence of street soccer (e.g., *Pelada*) to football training programmes and in fact, making the training curriculum even better by applying educational values to it.

Another contemporary trend of interest here is in relation to the practice of parkour, also known as free running, which involves the skillful negotiation of affordances of objects, surfaces, obstacles, gaps, ledges and inclines in the environment. As mentioned earlier, practice of parkour aligns well with the natural Brazilian way of playing. In a position paper, Strafford et al. (2018) provided insights on how parkour can act as a donor sport (i.e., various physical activities) for athletic development in youth team sports. They argue that “Integrating parkour-style activities into practice could develop/maintain athleticism and promote skill transfer in an enjoyable environment in team sport athletes due to utilization of performance-enhancing affordances and adaptive, functional, goal-directed movements” (p. 1). Moreover, Strafford et al. (2020) explored the views, experiences and insights of expert parkour-traceurs in relation to the enhancement of physical, cognitive, and perceptual skills through the practice of parkour. In line with their position paper, these expert participants concluded that parkour-style training environments indeed offer affordances (opportunity for actions) that enhance dynamic athletic performance in different sports, especially team sports like football (Strafford et al., 2020).

Therefore, the idea of parkour as a donor sport can be aligned with the unconventional learning environment to which many Brazilian children are exposed when engaged in all sorts of physical activities. This experience, in turn, can result in the enhancement of perceptual-motor skills of Brazilian football players as a function of playing under different environmental and task constraints. For this reason, design of parkour installations and open play areas can contribute to landscapes of varied affordances for physical activity and sports participation in urban settings such as favelas, inner city areas, and banlieues. These environmental constraints would provide means for people to entertain themselves inexpensively, gain access to employment opportunities and maintain health and well-being through (unstructured and more structured) sport and physical activities in dense urban environments.

### Final Remarks

From a sociological point of view, it has been argued that sport participation is highly dependent on the social class in which a child belongs. The poorer the child, the less likely she/he will have the means and the opportunity to participate in organized sport. Beyond sport participation, the problem of *Poverty* is further aggravated when it comes to health and educational related issues. Research has shown that *Poverty* in childhood may lead to higher risk of depression, substance abuse and other diseases in adulthood (see Costello et al., 2003). In addition, evidence suggests that growing up poor has long been associated with decreased educational accomplishment and lower earnings in lifetime. However, for those who receive some form of assistance, they are prone to overcome behavioral and emotional problems and in turn may have a better quality of life in the future (see Velasques-Manoff, 2014).

In contrast, as we have articulated in this article, from a skill acquisition point of view *Poverty* creates different contextual sensitivities that can positively influence the development of perceptual-motor skills in many children. These suggestions were supported by data from interviews, reports, and observations of the experiences of many successful Brazilian football players. However, being raised under precarious socio-economic conditions does not mean that every child will grow up strong, since each individual can respond in a different way to these conditions. Moreover, environmental constraints are very dynamic and change constantly as the norms and behavioral patterns in different societies are in constant transformation. Unfortunately, many of these alterations may negatively affect a society such as the case of misgoverned increase of urbanization without any sustainable and eco-friendly plans.

For these reasons, at the exosystemic level of a society, governors, and sport managers/administrators in contemporary societies should carefully consider the values and benefits of sport, play, recreation, and exercise participation. It is important to create means and opportunities for all citizens to engage with these activities, to promote physical and mental health and well-being as well as for the development of perceptual-motor skills for competitive sport performance. One way of accomplishing this important goal is through the means of designing and providing adequate *Pelada* and *Parkour* parks around dense urban environments such as inner-city areas, favelas, and banlieues. For example, in urbanized inner city London this aim has been documented in the creation of cage areas for football participation (see Smith, 2017; Kershaw, 2020).

## Data Availability Statement

The raw data supporting the conclusions of this article will be made available by the authors, without undue reservation.

## Ethics Statement

The studies involving human participants were reviewed and approved by the University of Otago, NZ. The patients/participants provided their written informed consent to participate in this study.

## Author Contributions

All authors listed have made a substantial, direct and intellectual contribution to the work, and approved it for publication.

## Conflict of Interest

The authors declare that the research was conducted in the absence of any commercial or financial relationships that could be construed as a potential conflict of interest.

## Footnotes

^1^The favela Vila Ana no longer exists as the area has been urbanized since conducting our fieldwork in this location (see Samora and Jimenez, 2019). For a brief overview of the ex-favela Vila Ana, see YouTube: https://www.youtube.com/watch?v=Y5h03ZQloSw. The futsal court, however, remained (see the Google maps link: https://goo.gl/maps/AuiX8N3EdQ6fSG6r9).
